# EFL learners’ problematic use of social media usage, classroom anxiety, perfectionism, and language attainment: correlations and perceptions

**DOI:** 10.1186/s40359-023-01419-5

**Published:** 2023-12-18

**Authors:** Juan Shu

**Affiliations:** College of Humanities, Ningbo University of Finance & Economics, Ningbo, 315175 China

**Keywords:** Language achievement, Chinese English language learners, Foreign language anxiety, Perfectionism, Problematic use of social media

## Abstract

**Background:**

The effect of social media (SM) on university students' educational progress and mental health has been studied in various contexts. However, the correlation between Chinese EFL learners' use of SM (problematic and non-problematic) and their language achievement, foreign language anxiety, and perfectionism requires further investigation.

**Objectives:**

To address this gap, a mixed-method research design was utilized. This study recruited 480 English language learners from Ningbo University of Finance and Economics in China using convenience sampling.

**Method:**

SM usage questionnaires, a perfectionism scale, a foreign language anxiety scale, self-assessment grids (CEFR) developed by the Council of Europe, and an interview checklist were used to collect data. Descriptive statistics (mean and SD) and Pearson correlation coefficient for each question were analyzed using SPSS. Thematic analysis was used to analyze the interviews.

**Results:**

The results revealed that problematic use of social media is associated with several negative consequences, such as low language achievement, high foreign language anxiety level, high academic burnout, and negative aspects of perfectionism.

**Conclusions and implications:**

These findings have theoretical and practical implications for language learners and teachers. The results can inform language educators about the potential negative effects of problematic SM use on language learning outcomes, mental health, and well-being. It is important to raise awareness of problematic SM use and to promote healthy SM habits among language learners. Additionally, the study highlights the importance of promoting the non-problematic use of SM as a potential resource to enhance language learning outcomes.

**Supplementary Information:**

The online version contains supplementary material available at 10.1186/s40359-023-01419-5.

## Introduction

Social networks play a pivotal role within educational environments, serving as a crucial communication channel and a wellspring of social support [1]. Several social networking platforms, such as Edmodo, are expressly crafted for educational purposes [2]. The educational advantages of social networks are manifold. They offer extensive access to information and informational resources, diminish barriers to group interactions and telecommunications [3], facilitate collaborative learning endeavors [4], stimulate learners to engage in self-directed learning [5], bolster engagement and learner motivation [6], foster interactivity between learners and their instructors [7], and promote active and social learning [5]. In sum, the advent of new technologies like the internet and social networks, while expanding opportunities for enhanced global communication, concurrently poses certain threats [8]. If not managed prudently, the misuse of social networks can yield negative repercussions on both the individual and societal levels. One such consequence is social networking addiction, a modern manifestation of soft addiction [9].

Various theories have been proposed to elucidate the roots of internet and social network addiction. Key theories encompass dynamic psychology theory, social control theory, behavioral explanations, biomedical explanations, and cognitive explanations. According to dynamic psychology theory, the origins of social networking addiction can be traced back to psychological trauma or emotional deficits in childhood, individual personality traits, and one's psychosocial status. Social control theory posits that the prevalence of addiction varies across demographics, such as age, gender, economic standing, and nationality, with specific addictions being more prevalent in certain societal segments than others [10]. Behavioral explanation theory asserts that individuals resort to social networks as a means of seeking rewards, such as escapism and entertainment. In accordance with biomedical explanation theory, the presence of particular chromosomes, hormones, or deficiencies in certain brain-regulating chemicals can contribute to addiction [11, 12]. Finally, cognitive explanation theory posits that social networking addiction arises from flawed cognitive processes, as individuals employ social networks to escape internal and external challenges [13]. Overall, social networking addiction is classified as a form of cyber-relationship addiction [14].

Social networking addiction denotes a preoccupation with social network use and the allocation of time to such networks in a manner that impairs other aspects of an individual's life, including professional and social engagements and overall well-being [9], culminating in life disruptions [10].

Social networking exerts adverse effects on both physical and psychological health, giving rise to behavioral disorders [15], depression [16, 17], anxiety, and manic symptoms [18]. For instance, a 2017 study on German students established a positive association between Facebook addiction and narcissistic traits, depression, anxiety, and stress [19]. It is posited that social networking addiction is more prevalent among individuals grappling with anxiety, stress, depression, and low self-esteem [4]. Griffith [20] characterizes addictive behavior as behavior manifesting specific attributes such as salience, mood modification, tolerance, withdrawal symptoms, conflict, and relapse [21]. Addictive behaviors are characterized by repeated habits that heighten the risk of diseases or social problems. Over the past decade, addictive behaviors, including internet and social network overuse, have become an integral facet of students' daily lives. Social networking addiction entails characteristics such as neglecting real-life challenges, self-neglect, mood fluctuations, concealing addictive behaviors, and experiencing mental distress [4].

In this context, signs and symptoms of social networking addiction encompass disruptions in daily tasks and activities, devoting more than an hour daily to social networks, a compulsion to inspect the profiles of former acquaintances, neglecting work and everyday responsibilities due to social network usage, and experiencing anxiety and stress stemming from a lack of access to social networks [22].

It is evident that multiple factors contribute to internet and social network addiction, including online shopping, dating, gaming, entertainment, utilizing mobile devices for internet access, searching for explicit content, individual personality traits, and low self-esteem [9, 21, 23–25].

Students represent a significant demographic within the virtual realm and social networks. The excessive use of social networks yields both positive and negative consequences on students' academic, social, and physical well-being [24]. A notable adverse outcome of overindulgence in social networks is diminished academic performance among students. Research conducted on medical students, for instance, revealed that students who exceeded the average levels of social network and internet use exhibited subpar academic achievements and a reduced ability to concentrate in the classroom [25]. Similarly, a study involving Qatari students demonstrated that students addicted to social networking displayed lower Grade Point Averages (GPAs) than their peers [26]. Findings from an Indian study underscored the negative effects of internet and social network addiction on students' academic performance and mental health [27, 28]. A Korean study unveiled a negative correlation between non-academic internet usage and students' academic performance [29]. Lastly, research conducted in Iran in 2018 disclosed a significant link between internet addiction and educational burnout [30].

Furthermore, social media (SM) platforms have been integrated into the realm of English language education. Researchers in the field of Second Language Acquisition (SLA) have conducted investigations into the impact of SM on English language education, highlighting the beneficial effects of SM usage on English proficiency, reading skills, writing style, communication skills, listening abilities, and grammatical usage for language learners [31–33]. Nonetheless, despite these advantages, there is a growing body of evidence indicating that excessive use of SM can lead to feelings of exhaustion [34]. The phenomenon of information overload and stress-related states stemming from SM-induced stress, along with user fatigue, is becoming increasingly prominent, particularly on platforms such as instant messaging and Facebook [35–38]. Online media can encroach upon users' lives [38], and an overabundance of user posts and SM-related inquiries has been associated with stress, irritability, and sentiments of being overwhelmed and fatigued [39, 40]. These negative perceptions and emotional issues are often exacerbated by extended internet usage across various devices and applications. For instance, smartphones are consistently within reach [41], and emails can be accessed through various devices, including desktop computers and mobile phones [38].

Despite the challenges and adverse outcomes linked to SM usage, it is undeniable that online discussions and written interactions have supplanted traditional face-to-face conversations, contributing to the enhancement of users' language skills. Prolonged utilization of cell phones, tablets, and laptops equipped with SM applications has exposed users to rich linguistic input, thus augmenting their capacity to generate coherent output in a second language (L2) [42]. However, as students increasingly opt for non-traditional teaching methods and approaches, research on the effects of SM on learning and education, especially in the context of English as a foreign/second language (EFL/ESL), remains relatively limited. Consequently, this study seeks to investigate the impact of SM on EFL learners' anxiety, perfectionism, foreign language anxiety, and language proficiency, as well as to explore their perceptions of problematic SM usage. The research also delves into the perspectives of EFL learners regarding SM usage. To address these objectives, the following research questions have been formulated.

1. Is there any statistically significant correlation between Chinese EFL learners’ problematic social media usage and foreign language classroom anxiety?
2. Is there any statistically significant correlation between Chinese EFL learners’ problematic social media usage and their perfectionism state?
3. Is there any statistically significant correlation between Chinese EFL learners’ problematic social media usage and their language achievement?
4. How do Chinese EFL learners perceive problematic use of social media usage in their educational and personal life?

### Review of the related literature

This study mainly focuses on language learners' use of SM, foreign language anxiety, perfectionism, language achievement, and academic burnout. The studies on each variable are reviewed separately in the following sections**.**

### Social media and EFL learners’ language anxiety

Anxiety is a distressing feeling associated with a current traumatic situation or anticipation of news, dependent on an uncertain object. It is a concept of threat or insecurity expressed differently by different people [43]. However, a review of the related literature shows conflicting results. For instance, Jiang [44] stated that there is a strong correlation between problematic SM usage among Chinese university students and their level of anxiety. Similarly, Andreassen and Pallesen [45] found that when students have a strong incentive to use SM and spend too much time using the internet and SM, they encounter impairments in their professional, personal, and social life, as well as mental health and well-being.

Similarly, Lepp et al. [46] reported a strong correlation between SM use and anxiety levels. In another related study, it was found that particularly during the pandemic when people had to use SM, they consciously and unconsciously received negative information, such as fake news, which in turn increased anxiety levels among the people, especially frequent users of SM [47]. Thorisdottir et al. [48] studied SM use and anxiety disorder among Icelandic adolescents in another context. They reported a positive correlation between SM usage and anxiety symptoms for male and female university students. Similarly, Wong et al. [49] and Hussain and Griffiths [50] found that the association between anxiety and problematic SM usage is positively significant.

### Social media and perfectionism

Perfectionism is a personality characteristic that reflects an individual's high-performance standards and high critical self-evaluation [51–54]. Previous studies [55, 56] indicate that perfectionism may appear at any age and might influence a person's success and failure. However, perfectionism has been associated with various psychopathological phenomena, including anxiety, depression, obsessions, and psychosomatic disorders [57, 58]. Sarafraz et al. [57] have also argued that perfectionism is a component of some personality disorders. Related studies have also revealed that SM usage might impact individuals' perfectionism state [44].

Furthermore, studies indicate that perfectionism in SM might be linked to different domains, such as parenting [59], physical appearance [46], and other domains [59]. In another study, Casale et al. [60] reported a positive correlation between internet addiction and perfectionism. Sedera and Lokuge [42] introduced digital perfectionism as a new mental disorder caused by technology. Haren et al. [56] explored the association between problematic SM usage, perfectionism, online cognitions, metacognitions, and SM behavior to explore the factors affecting SM behavior. They reported a significant correlation between inappropriate SM usage and perfectionism.

### Social media and language achievement

Social media (SM) refers to any content-based form of e-communication that enables users to share information and ideas [61]. SM has been widely used in different fields, especially in EFL/ESL education [61]. Several studies have reported the potential benefits of SM in language learning, such as enhancing pronunciation accuracy, developing vocabulary, and promoting confidence in speaking English [62, 63]. SM has also been found to improve collaborative learning, teamwork, critical thinking, and literacy proficiency among language learners [64, 65]. Furthermore, SM has been reported to foster student engagement and motivation in tertiary education, with faculty members incorporating SM into their classes [66]. SM has also been used by students to communicate, learn, and meet their educational needs [67]. SM has been found to help teachers stay connected and learn more about students' engagement in classroom assignments [68]. Moreover, younger faculty members tend to use SM more frequently for educational and academic purposes [69]. SM applications such as Facebook have also been employed for pedagogical activities to foster language use at home and outside the classroom [70].

In summary, the research on the use of SM in teaching and learning EFL/ESL has become widespread, and the findings suggest that SM has a positive impact on language learning and teaching. SM platforms are believed to be beneficial to support pedagogical purposes, fostering English skills, developing social skills, and promoting active learning. Therefore, the potential of SM in language learning and teaching should be further explored and utilized to enhance the quality of education.

## Materials and methods

### Participants

Two groups of participants were recruited: participants for the quantitative phase and informants for the qualitative phase. The participants were selected from Ningbo University of Finance and Economics, Ningbo, China. For the quantitative phase, 480 EFL learners were selected through convenience sampling during the spring semester of 2021. Although 600 undergraduates from Ningbo University of Finance and Economics, were recruited for the quantitative phase, only 480 returned the questionnaires. Participants' self-reports revealed that they were all native speakers of Chinese, aged between 18 and 27, with 230 female and 250 male participants. Only the participants who were taking English language courses were selected. All participants were informed of the study's purpose and filled in informed consent forms. They were also assured that their responses to the questionnaires would not affect their academic achievement at the university, and the collected data would be kept confidential.

Among the participants recruited for the quantitative phase, 30 language learners were nominated and invited for the qualitative phase. However, due to data saturation occurring after the 20th informant was interviewed, only 20 were ultimately interviewed. The informants were selected through theoretical sampling.

### Research method

A mixed-methods research design (explanatory sequential research method: Quan-Qual) was utilized to answer the research questions. The ex-post facto (correlational) research method was employed to investigate research questions 1, 2, 3, and 4, measuring variables such as problematic and non-problematic SM usage, academic burnout, foreign language anxiety, and perfectionism. All variables were measured quantitatively using interval scales. For the qualitative phase, a phenomenology research method was employed to deeply explore the lived experiences of those engaged in and affected by the phenomenon. Phenomenology is commonly used by researchers interested in investigating fields with little or no prior knowledge. Participation in the qualitative phase was voluntary, and individuals received an initial explanation of the study's procedures before deciding to participate.

### Data analysis

The data analysis was conducted using MAXQDA software (version 2022) as recommended by Creswell [71] The unit of analysis was the sentence, and the researcher analyzed manifest content rather than latent content. The qualitative data were collected, analyzed, and reported in English. An inductive approach to content analysis was taken, as no theory or framework guided the generation of codes, categories, and themes [52]. Gao et al.’s [58] five sequential steps for qualitative data analysis were followed. Firstly, the data were cleaned up by correcting linguistic errors, ambiguities, inaccuracies, or repetitions. Secondly, the researcher read the data multiple times and developed open codes. Thirdly, the open codes were categorized as relevant axial codes/subtopics. Fourthly, the axial codes/subtopics were grouped under higher-order selective codes/general themes. Lastly, a detailed and complete report was prepared on the completed process of data analysis and its interpretation.

For the generated codes, topics, and categories, their frequency was reported, and the results were presented visually using the MAXMAP properties of MAXQDA. To ensure the credibility of the analytical process, 20% of the generated codes were randomly selected and re-coded by a second coder who was a university lecturer in applied linguistics with sufficient knowledge and experience in conducting qualitative research studies. Specifically, 100 codes were created in this study, and 20 of them were sent to the second coder. The intercoder agreement coefficient for this study was 96%, but the second coder disagreed with the first coder on one code. The two coders discussed and resolved the disagreement to complete the qualitative data process.

### Measures

To answer the research questions, we used different measures. Each is explained as follows.

### Foreign Language Anxiety (FLA) scale

The assessment of various components in this study was carried out using specific instruments and measures. First, to evaluate participants' Foreign Language Anxiety (FLA), the instrument validated by Zhao [72] was employed. This questionnaire comprises 33 statements, which were categorized into four components: communication anxiety (8 items), negative evaluation (9 items), test anxiety (5 items), and anxiety concerning English classes (11 items). The internal consistency and subscales of the questionnaire were estimated using Cronbach’s alpha. The obtained Cronbach’s alphas ranged from α = 0.82 to 0.87, indicating that the FLA scale exhibited acceptable reliability across its components as well as for the overall scale.

Second, the Problematic Mobile Social Media Usage Assessment Questionnaire [54] was utilized to gauge participants' usage patterns. The problematic SM usage scale, developed by Jiang in 2018, comprises 20 items rated on a 5-point Likert-type scale (1 = inconsistent, 5 = totally consistent) with no reverse scoring. Scores on all items were summed, and higher scores indicated more problematic use. The reliability of both problematic and non-problematic usage components was assessed using Cronbach's alpha, revealing high reliability (0.86 and 0.82, respectively). Third, perfectionism among the participants was assessed using the Chinese version of the Multidimensional Perfection Scale (MPS) developed by Dai [73]. This instrument consists of 29 items categorized into two subscales. The first component, referred to as Perfectionism High Standard, encompasses 15 items, while the second component, known as Perfectionism Adaptability, comprises 14 items. Each item is rated on a 5-point scale (1 = very much disagree, 2 = somewhat disagree, 3 = no opinion, 4 = somewhat agree, 5 = very much agree). The score on the high standard section reflects an individual’s inclination toward perfectionism, whereas the score on the adaptability component indicates the degree of maladjustment. Both sections exhibited an acceptable level of internal consistency (α = 0.85 for high standards; α = 0.87 for adaptability).

To assess participants' language achievement, the Self-assessment Grids based on the Common European Framework of Reference for Languages (CEFR) developed by the Council of Europe were employed. Participants were provided with the rubric and explanations of each scale in the instruction section. They were asked to rate their language proficiency using the CEFR Self-assessment grid, providing a numerical equivalent for their language skills. The reliability of the scoring was established by asking participants to rate their language proficiency twice, with the correlation coefficient between the two sets of scores demonstrating good reliability (*r* = 0.86).

The final instrument employed in this study was an interview checklist, encompassing individual face-to-face interviews, phone interviews, and online interviews. Participants were given the option to respond to the questions in either English or Chinese. The interview checklist comprised open-ended questions designed to capture the interviewees' perspectives on both the favorable and adverse outcomes of problematic and non-problematic social media (SM) usage. For example, interviewees were prompted to identify and elucidate potential positive and negative impacts of SM applications like Telegram, WhatsApp, and others, on various aspects such as their language achievement, mental well-being, anxiety levels, and academic attainment.

### Data analysis procedure

The data analysis procedures encompassed a variety of techniques. Quantitative data were subjected to analysis employing SPSS, wherein mean scores and standard deviations were computed for each query. Additionally, the correlation coefficient between variables was determined at a significance threshold of *p* = 0.05. Conversely, the data pertinent to research question 5 were subjected to analysis utilizing MAXQDA software (version 2022. The unit of analysis was the sentence, with the researcher focusing on the analysis of manifest content, rather than latent content. The qualitative data were gathered, analyzed, and reported in the English language. An inductive content analysis approach was pursued, as there was an absence of a guiding theory or framework for the generation of codes, categories, and themes, following Berg's approach [74]. The qualitative data analysis adhered to Gao et al.’s [58] five-step sequential process. Initially, the data underwent a cleansing process, addressing linguistic errors, ambiguities, inaccuracies, or repetitions. Subsequently, the researcher meticulously reviewed the data multiple times and formulated open codes. These open codes were then organized into relevant axial codes or subtopics. These axial codes or subtopics were further clustered under overarching selective codes or general themes. Finally, a comprehensive and detailed report was compiled, delineating the completed process of data analysis and its interpretation.

To ensure the credibility of the analytical process, 25% of the generated codes underwent a random selection for re-coding by a second coder who held the position of a university lecturer in applied linguistics. This second coder possessed substantial knowledge and experience in conducting qualitative research studies. In this study, a total of 40 codes were generated, with 10 of them being reviewed by the second coder. The intercoder agreement coefficient for this study was established at 90%. However, a single instance of disagreement between the two coders surfaced, necessitating a thorough discussion and resolution of this discrepancy to conclude the qualitative data processing.

## Results

### Quantitative findings

The quantitative results are presented in Tables 1 and 2. Then, the results for each question are presented and discussed sequentially.

**Table 1:** Demographic profile of the respondents selected for quantitative and qualitative phases

Phase	Variable	Number (%)
Quantitative	Gender	
	Male	230 (41.2%)
	Female	250 (52.8)
	Age	
	18–20	60 (12.5%)
	21–23	230 (48%)
	24–27	190 (39.5%)
Qualitative	Gender	
	Male	10 (50%)
	Female	10 (50%)
	Age	
	18–20	6 (30%)
	21–23	7(35%)
	24–27	7 (35%)

**Table 2:** Means, standard deviations, and bivariate correlations between variables

Variables	Mean	SD	1	2	3	4
(1) Problematic use of S.M	65	15	1			
(2) Foreign language anxiety	75	17.5	0.63**	1		
(3) Language achievement	71	-0.24	0.42***	-.45**	1	
(4) Perfectionism	79	20	0.44**	0.17	0..42**	1

^*^p < 0.05, **p < 0.01, ***p < 0.001, l tests were two-tailed. (*n* = 590)

As seen in A significant positive correlation exists between the language learners' problematic use of social media (M = 65, SD = 15) and foreign language anxiety.

Language learners, on average, report a moderate level of foreign language anxiety (M = 75, SD = 17.5). Furthermore, there is a noteworthy and statistically significant positive correlation between foreign language anxiety and their problematic use of social media.

Language achievement among the participants is moderately rated, with a mean of 71 (SD = 24). It's noteworthy that a statistically significant positive correlation exists between foreign language anxiety and language achievement, as well as a statistically significant negative correlation between problematic use of social media and language achievement.

Participants in the study exhibit a moderate level of perfectionism (M = 79, SD = 20). Notably, there are statistically significant positive correlations between perfectionism and both foreign language anxiety and problematic use of social media.

### Research question 1

The study utilized Pearson product-moment correlation analysis to examine the relationship between participants' non-problematic social media (SM) usage and their levels of foreign language anxiety (FLA). A significant positive correlation exists between the language learners' problematic use of social media (M = 65, SD = 15) and foreign language anxiety. Language learners, on average, report a moderate level of foreign language anxiety (M = 75, SD = 17.5). Furthermore, there is a noteworthy and statistically significant positive correlation between foreign language anxiety and their problematic use of social media. The simple regression model produced an R^2^ value of 0.70, F (2, 477) = 104, *p* < 0.001. As shown in Table 3, the problematic use of SM had significant positive regression weights, indicating that language learners with higher scores on problematic SM usage were expected to have higher FLA.

**Table 3:** Results from the regression analysis (SM usage and FLA)

Model		Unstandardized Coefficients		Standardized Coefficients	t	Sig
		B	Std. Error	Beta		
1	(Constant)	60.12	8.25		7.23	.001
	PSMU	.622	.083	0.55	7.52	.001

*NPSMU* non-problematic social media usage, *PSMU* problematic social medial usage

### Research question 2

The study used simple regression and correlation analyses to investigate the relationship between participants' language achievement and non-problematic social media (SM) usage. Table 1 presents the descriptive statistics and analysis results. The results show (Table 4) that, problematic SM usage was negatively correlated with participants' language achievement (*r* = -0.38, *p* < 0.001).

**Table 4:** Results from the regression analysis (SM usage and language achievement)

Model		Unstandardized Coefficients		Standardized Coefficients	t	Sig
		B	Std. Error	Beta		
1	(Constant)	30.157	9.490		3.178	.002
	PSMU	-.034	.088	-.034	-.386	.700

### Research question 3

The study utilized Pearson product-moment correlation analysis to examine the relationship between participants' non-problematic social media (SM) usage and perfectionism. The results revealed that problematic SM usage was significantly positively correlated with participants' perfectionism (*r* = 0.46, *p* < 0.001). However, the correlation between non-problematic SM usage and perfectionism was significantly negative (*r* = -0.30, *p* < 0.01). As shown in Table 5, problematic use of SM had a significant positive regression weight, indicating that respondents with higher scores on problematic SM usage had a higher level of perfectionism.

**Table 5:** Results from the regression analysis (SM usage and perfectionism)

Model		Unstandardized Coefficients		Standardized Coefficients	t	Sig
		B	Std. Error	Beta		
1	(Constant)	48.718	7.698		6.866	.001
	PSMU	.600	.091	.349	7.440	.001

### Research question 4

#### Qualitative findings

The interviews with 20 Chinese learners of the English language were transcribed word-by-words. Then, they were analyzed using axial and open codlings. Two principal codes were extracted, explained, and exemplified in the following sections.

##### SM causes addiction

Despite its advantages, the pervasive use of SM has prompted concerns about addiction among participants. Many express a sense of irresistible compulsion to frequently engage with SM, often diverting their attention from important daily activities. Participant 3, for example, admits to checking SM applications even during study sessions, describing it as uncontrollable behavior. Participant 9 similarly mentions the frequent and distracting use of SM throughout the day.

##### SM causes loneliness

A significant number of participants highlight the paradox of increased SM use leading to feelings of loneliness and isolation. Time spent on SM detracts from opportunities for face-to-face interactions with family and friends, thereby contributing to mental health issues like depression and anxiety. Participant 7 elucidates how excessive SM use diminishes their connection with loved ones and fosters a sense of isolation from society and family.

##### Social media enhances academic burnout

SM is associated with increased academic burnout among language learners. Interviewees point out that excessive SM usage can lead to reduced academic performance, as it diverts time and attention away from language studies. Participant 6 underscores the attraction of SM content in the Chinese language over English, which affects motivation for language learning.

##### Social media enhances social anxiety disorder

The utilization of SM can exacerbate social anxiety disorders. Participants often compare their online personas to real-life interactions, leading to anxiety in face-to-face situations. The lack of in-person communication contributes to this anxiety, as expressed by Participant 13.

##### SM leads to grammar and spelling errors

Some interviewees notice a proliferation of typos and intentionally incorrect English usage in posts, comments, and texts on SM applications. This casual approach to language usage, as exemplified by Participant 9, results in frequent lapses in punctuation and spelling.

##### SM increases foreign language anxiety and reduces self-efficacy

Exposure to SM content created by native speakers can heighten anxiety among language learners about attending English language classes and speaking in real-life scenarios. This comparison often results in reduced self-efficacy, as articulated by Participant 7.

##### SM leads to inequality in education

Participants note that access to the internet and SM is essential for educational resources and language skill improvement. This creates inequalities, as those without access to SM and the internet are left at a disadvantage. Participant 3 exemplifies how low internet speeds affected their performance in an English course. This comprehensive analysis underscores the intricate landscape of SM usage among language learners, acknowledging its merits while highlighting the critical challenges and pitfalls that demand thoughtful consideration within pedagogical contexts.

## Discussion

The outcomes of this study shed light on the correlation between problematic social media (SM) usage and Foreign Language Anxiety (FLA) among Chinese language learners. This finding is consistent with prior research that has established connections between excessive SM engagement and adverse effects such as compromised sleep quality, diminished self-esteem, heightened anxiety, and increased depression [57]. It is posited that the pervasive use of SM may displace in-person social interactions, thereby eroding social capital, fostering feelings of depression and solitude, and ultimately exacerbating anxiety, including FLA.

The findings of this investigation align with the results reported by Jiang [44], who identified that Chinese students who spend more time on SM tend to report elevated anxiety levels. Additionally, other scholars [15] have demonstrated that SM utilization can incite stress-related concerns, including fatigue, particularly in the context of platforms like Facebook and instant messaging. It is proposed that excessive exposure to other users' comments, posts, and tweets can evoke irritation, stress, feelings of overwhelm, and fatigue, culminating in heightened anxiety.

A plausible explanation for the positive correlation between problematic SM usage and anxiety is rooted in the potential for SM and internet addiction. Yang and Wu et al. [75, 76] posit that greater SM and internet use correspond to an increased risk of addiction. Similarly, Simsek and Sali [77] have identified that individuals using SM for social communication are more likely to develop SM addiction compared to those who employ it for recreational purposes. The consequential lifestyle habits associated with excessive SM use, characterized by a loss of control and a sense of isolation, are known to deplete psychological capital [60].

Conversely, the negative correlation between non-problematic SM usage and anxiety may stem from the fact that individuals using SM for informative, entertaining, and social purposes tend to possess high levels of social capital. In these communities, members find substantial support and do not experience feelings of loneliness or depression. Furthermore, the positive correlation between SM use and participants' language performance and language skill development aligns with Wang and Vasquez's findings [78]. Their examination of the effects of Facebook usage on language learning identified a significant difference in writing quantity between the experimental group using Facebook and the control group, although not in writing quality. It was recommended that Facebook be employed for extracurricular activities to enhance learners' writing abilities. Another justification for the correlation between non-problematic SM usage and language learning is rooted in the findings of Jafari et al. [79], who observed notable individual differences between students who used Facebook and those who did not. SM applications provide an additional source of comprehensible input that users can engage with at their own pace, thereby bolstering language learners' interaction and communication skills.

The results of this study align with previous research by Jafari et al. [79] and Kumar et al. [80] both of which identified a negative association between excessive internet use and academic performance. Likewise, Fatehi et al. [63] reported that students with excessive internet and SM use tended to achieve lower GPAs compared to those with regular use. Furthermore, Jafari et al. [79] established a significant correlation between internet addiction, academic burnout, and performance among nursing students. Mei et al. [64] identified connections between health, self-control, self-esteem, and problematic internet and social media use among Chinese high school students. Similarly, Peterka-Bonetta et al. [65] uncovered positive relationships between depression and internet use disorder and burnout and internet use disorder. Berte et al. [66] documented a negative correlation between university students' perceived self-efficacy and excessive internet use.

The findings of this study corroborate the work of Harren et al. [67] who unearthed a significant correlation between problematic SM use and perfectionism. The excessive use of SM is believed to induce stress and emotional exhaustion, both of which are associated with perfectionism [32, 36]. SM users may engage in social comparisons, which can engender feelings of inadequacy and fuel perfectionism [32, 36]. Existing research has illustrated that SM platforms and applications facilitate self-comparisons, particularly in domains like appearance, health, and parenting, which can serve as predictors of perfectionism [27]. Additionally, the correlation between perfectionism and problematic SM usage can be ascribed to SM users' engagement in social comparisons and self-presentation, culminating in the proliferation of social comparisons and, ultimately, perfectionism.

The study's findings underscore the positive impact of appropriate SM and internet usage on English as a Foreign Language (EFL) learners' language acquisition. This impact manifests through exposure to authentic materials and the abundance of opportunities to practice language skills and sub-skills. This finding resonates with the research conducted by Noori et al. [68], which documented the frequent use of SM applications like Facebook, WhatsApp, and YouTube in English language education. Salih and Elsaid [81] similarly concluded that SM represents an effective technological tool for EFL instruction.

Nonetheless, this study reveals several negative consequences associated with SM use in language learning, including SM addiction, grammar errors, social anxiety, language anxiety, educational disparities, academic burnout, and loneliness. These findings align with the work of An and Williams [69], who found that exposure to SM can make some language learners self-conscious, and with Mitchell [70], who reported that some students were concerned with the spelling and grammar errors they encountered while reading SM posts. Bani-Hani et al. [82] also noted that students were distracted by instant messaging and experienced anxiety as they attempted to match their peers' language proficiency. In conclusion, this study underscores that while SM usage can have both beneficial and detrimental impacts on EFL learners' language acquisition, the judicious and discerning use of SM and the internet is paramount to optimizing the benefits while mitigating negative repercussions. These adverse effects are corroborated by Noori et al. [68], who found that "75% of the participants believed that the overuse of social media caused eye problems for both lecturers and students" (p.11). This aligns with the results of Haand and Shuwang's study [83] which revealed that excessive SM use is linked to health and mental health problems among students and teachers [73–91].

## Conclusions

In conclusion, this study has provided valuable insights into the intricate relationship between problematic social media (SM) usage and Foreign Language Anxiety (FLA) among Chinese language learners. The findings indicate a positive correlation, suggesting that excessive engagement with SM platforms may contribute to heightened FLA. These results align with previous research emphasizing the adverse consequences of excessive SM use, such as compromised sleep quality, diminished self-esteem, and increased anxiety and depression. Moreover, the study underscores the significance of the purpose and nature of SM engagement. Non-problematic SM usage, which prioritizes informative, entertaining, and social objectives, demonstrates a negative correlation with anxiety. Language learners who harness SM for constructive purposes appear to foster social capital, consequently alleviating feelings of loneliness and depression.

### Implications

The implications of this study are twofold. Firstly, educators and language instructors should be cognizant of the potential impact of SM usage on language learners' psychological well-being. Recognizing the risk of excessive SM usage contributing to FLA and anxiety-related issues, educational institutions may consider implementing interventions and educational programs to promote balanced and purposeful SM usage among students. This includes guiding learners to employ SM for educational and informative purposes, fostering a positive learning environment [80, 92].

Secondly, this study underscores the importance of informed and purposeful SM engagement. Learners and SM users should be educated about the potential consequences of their SM usage habits, encouraging them to make intentional choices in their online activities. Such awareness can promote healthy digital behavior and diminish the risks of social media-related anxiety.

### Limitations

Several limitations of this study must be acknowledged. Firstly, the research primarily relied on self-reported data from participants, which may be influenced by social desirability bias and subjectivity. Furthermore, the study did not examine the impact of the duration and frequency of SM usage on anxiety levels, which might offer a more nuanced understanding. Additionally, the study concentrated on a specific context of Chinese language learners, potentially limiting the generalizability of the findings to broader populations. Further research with diverse cultural and linguistic backgrounds is essential to ascertain the broader applicability of the findings.

#### Areas for further studies

This study has laid a foundation for several areas that merit further exploration. Firstly, future research should employ more diverse and representative samples to better generalize the results. Investigating the influence of SM usage duration and frequency on anxiety levels would provide a more comprehensive understanding of this complex relationship.

Additionally, studies can delve deeper into the positive effects of non-problematic SM usage, not only in language learning but also in various educational contexts. The specific mechanisms and practices that enhance language skills and sub-skills through purposeful SM engagement warrant further investigation.

Exploring interventions and strategies for promoting balanced and constructive SM usage among learners is an imperative avenue for future research. These interventions can be designed to mitigate the adverse consequences and promote the beneficial aspects of SM usage in education and language learning. Lastly, expanding research into the impact of SM on language learners' well-being beyond FLA is crucial. This could encompass a broader examination of mental health, self-esteem, and overall life satisfaction among language learners who engage with SM.

### Supplementary Information

**Additional file 1. **

## Data Availability

The data will be made available by the authors without undue reservation.

## References

[1] Cadima R, Ojeda Rodríguez J, Monguet Fierro JM (2012). Social networks and performance in distributed learning communities. Educ Technol Soc.

[2] Manca S, Ranieri M (2017). Implications of social network sites for teaching and learning. Where we are and where we want to go. Educ Inf Technol..

[3] Madaiah M, Seshaiyengar CT, Suresh P, Munipapanna S, Sonnappa SD (2017). Study to assess the effects of social networking sites on medical college students. Int J Community Med Public Health.

[4] Al-Dhanhani A, Mizouni R, Otrok H, Al-Rubaie A (2015). Analysis of collaborative learning in social network sites used in education. Soc Netw Anal Min.

[5] Keleş E, Demirel P, editors. Using Facebook in Formal Education as a Social Network. 5th International Computer & Instructional Technologies Symposium; 2011.

[6] Hamid S, Waycott J, Chang S, Kurnia S (2011). Appropriating online social networking (OSN) activities for higher education: two Malaysian cases. Changing Demands, Changing Directions Proceedings ascilite Hobart.

[7] Hamid S, Waycott J, Kurnia S, Chang S (2015). Understanding students' perceptions of the benefits of online social networking use for teaching and learning. Internet High Educ.

[8] Avcı K, Çelikden SG, Eren S, Aydenizöz D (2015). Assessment of medical students’ attitudes on social media use in medicine: a cross-sectional study. BMC Med Educ.

[9] Schou Andreassen C, Pallesen S (2014). Social network site addiction-an overview. Curr Pharm Des.

[10] Zaremohzzabieh Z, Samah BA, Omar SZ, Bolong J, Kamarudin NA. Addictive Facebook use among university students. arXiv preprint arXiv:150801669. 2015.

[11] Sadock BJ, Sadock VA. Kaplan and Sadock's synopsis of psychiatry: behavioral sciences/clinical psychiatry: Lippincott Williams & Wilkins; 2011.

[12] Young KS, Rogers RC (1998). The relationship between depression and internet addiction. Cyberpsychol Behav.

[13] Beard KW (2005). Internet addiction: a review of current assessment techniques and potential assessment questions. Cyberpsychol Behav.

[14] Alavi SS, Jannatifard F. Internet addiction Definitions, Dimensions, Diagnosis: Isfahan University of Medical Science (Persian); 2012.

[15] Can L, Kaya N (2016). Social networking sites addiction and the effect of attitude towards social network advertising. Procedia Soc Behav Sci.

[16] Masthi NR, Pruthvi S, Phaneendra M (2018). A comparative study on social media usage and health status among students studying in pre-university colleges of urban Bengaluru. Indian J Community Med.

[17] Wang P, Wang X, Wu Y, Xie X, Wang X, Zhao F (2018). Social networking sites addiction and adolescent depression: a moderated mediation model of rumination and self-esteem. Personal Individ Differ.

[18] Tang CSK, Koh YYW (2017). Online social networking addiction among college students in Singapore: comorbidity with behavioral addiction and affective disorder. Asian J Psychiatr.

[19] Brailovskaia J, Margraf J (2017). Facebook addiction disorder (FAD) among German students—a longitudinal approach. PLoS ONE.

[20] Griffiths M (2005). A ‘components’ model of addiction within a biopsychosocial framework. J Subst Abus.

[21] Sun T, Wu G (2012). Traits, predictors, and consequences of Facebook self-presentation. Soc Sci Comput Rev.

[22] Krishnamurthy S, Chetlapalli SK (2015). Internet addiction: prevalence and risk factors: a cross-sectional study among college students in Bengaluru, the Silicon Valley of India. Indian J Public Health.

[23] Vahidi Far H, Nabavi Zadeh H, Ardebily FM (2014). Assessment of internet addiction among college students in North Khorasan University of Medical Sciences in Bojnoord, Iran. J North Khorasan Univ Med Sci.

[24] Chaudhari B, Menon P, Saldanha D, Tewari A, Bhattacharya L (2015). Internet addiction and its determinants among medical students. Ind Psychiatry J.

[25] Jha RK, Shah DK, Basnet S, Paudel KR, Sah P, Sah AK (2016). Facebook use and its effects on the life of health science students in a private medical college of Nepal. BMC Res Notes.

[26] Upadhayay N, Guragain S (2017). Internet use and its addiction level in medical students. Adv Med Educ Pract.

[27] Al-Yafi K, El-Masri M, Tsai R (2018). The effects of using social network sites on academic performance: the case of Qatar. J Enterp Inf Manag.

[28] Kumar S, Kumar A, Badiyani B, Singh SK, Gupta A, Ismail MB (2018). Relationship of internet addiction with depression and academic performance in Indian dental students. Clujul Med.

[29] Kim SY, Kim MS, Park B, Kim JH, Choi HG (2017). The associations between internet use time and school performance among Korean adolescents differ according to the purpose of internet use. PLoS ONE.

[30] Imani A, Esmaeeli S, Golestani M, Ghoddoosi-Nejad D, Baghban E. Relation between internet addiction and educational burnout among students in Faculty of Health Management and Medical Informatics of Tabriz University of Medical Sciences: a cross-sectional study. Modern Care J. 2018.

[31] Muftah M (2022). Enhancing EFL learners' reading and writing skills through social media: a case study. Engl Lang Teach.

[32] Kolhar MA, Hussain A, Raza SA (2021). Impact of social media on academic performance of students: a case study of Pakistan. J Educ Educ Dev.

[33] Wamba TF, Carter M (2016). The impact of social media on English language proficiency: a case study of undergraduate students at University of Buea. Int J Eng Lang Teach.

[34] Fu J, Wang Y, Chen X (2020). The effects of social media overload on college students' mental health: Evidence from China. J Educ Comput Res.

[35] Bright LF, Kleiser LR, Grau SL, Hamby JM (2015). Social media and college admissions: the first longitudinal study. J Mark High Educ.

[36] Dhir A, Yossatorn Y, Kaur P, Chen S (2018). Online social media fatigue and psychological wellbeing—A study of compulsive use, fear of missing out, fatigue, anxiety and depression. Int J Inf Manage.

[37] Han Y (2018). Why do people use Facebook? A uses and gratifications approach. Comput Hum Behav.

[38] Syvertsen T. The media welfare state: Nordic media in the digital era. Michigan: University of Michigan Press; 2020.

[39] Haren TV, Cotten SR (2021). Social media use and mental health: a review of the literature. Curr Opin Psychol.

[40] Shi J, Luo Y, Yen DC (2020). Understanding the effects of social media overload on users' subjective well-being: a longitudinal study. Int J Inf Manage.

[41] Barley SR, Meyerson DE, Grodal S, Eunyoung C (2011). Email as a source and symbol of stress. Organ Sci.

[42] Al Jahromi ZM (2020). The impact of technology on language learning and language learning strategies. J Lang Linguist Stud.

[43] American Psychological Association (2021). Anxiety.

[44] Jiang Z (2020). Social media use and academic burnout: the mediating role of academic procrastination. J Educ Comput Res.

[45] Andreassen CS, Pallesen S (2014). Social network site addiction—an overview. Curr Pharm Des.

[46] Lepp A, Li J, Barkley JE, Salehi-Esfahani S (2014). Exploring the relationship between college students’ cell phone use, personality, and leisure. Comput Hum Behav.

[47] Padoa A, Pellegrini M, Iafrate R (2018). Perfectionism in parenting and social media use: a study on Italian mothers. J Child Fam Stud.

[48] Thorisdottir IE, Sigurvinsdottir R, Asgeirsdottir BB, Allegrante JP, Sigfusdottir ID (2019). Active and passive social media use among Icelandic adolescents: the impact on symptoms of anxiety and depression. Scand J Psychol.

[49] Wong TKY, Law YK (2018). The effects of social media addiction on university students' academic performance: a case study in Hong Kong. J Educ Comput Res.

[50] Hussain Z, Griffiths MD (2018). Problematic social networking site use and comorbid psychiatric disorders: a systematic review of recent large-scale studies. Front Psych.

[51] Houkes I, Winants YH, Twellaar M, Verdonk P (2011). Development of burnout over time and the causal order of the three dimensions of burnout among male and female GPs. A three-wave panel study. BMC Public Health.

[52] Egan SJ, Wade TD, Shafran R (2011). Perfectionism as a transdiagnostic process: a clinical review. Clin Psychol Rev.

[53] Melero ME, Pino MJ, Quiles MN (2020). Perfectionism and academic achievement: the mediating role of academic self-efficacy and academic motivation. Front Psychol.

[54] Han Y (2017). The relationship between academic burnout and social media addiction among Chinese college students: a moderated mediation model. Curr Psychol.

[55] Jafari Z, Haghani F, Borji M (2022). The relationship between academic burnout and abusive internet use among nursing students. J Nurs Educ Pract.

[56] Haren MT, Valjavec-Gratian M, Tkalčić M (2021). The role of perfectionism, online cognitions and metacognitions in predicting social media behavior. Pers Individ Differ.

[57] Sarafraz MR, Ebrahimi A, Shokrpour N (2020). Perfectionism and personality disorders: a systematic review and meta-analysis. J Psychiatr Res.

[58] Gao J, Zheng P, Jia Y, Chen H, Mao Y (2020). Mental health problems and social media exposure during the COVID-19 outbreak. PLoS ONE.

[59] Stoeber J, Otto K (2006). Positive conceptions of perfectionism: Approaches, evidence, challenges. Pers Soc Psychol Rev.

[60] Casale S, Rugai L, Fioravanti G (2014). Perfectionism and internet addiction: a study on Italian university students. Pers Individ Differ.

[61] Al-Khalidi A, Khouni L (2021). The use of social media in EFL/ESL education: a systematic review of empirical studies. Educ Inf Technol.

[62] Encalada MAR, Sarmiento HGH (2019). The use of social media in EFL learning and teaching: a review of empirical studies. PROFILE Issues Teach Prof Dev.

[63] Fatehi F, Monajemi A, Sadeghi A, Mojtahedzadeh R, Mirzazadeh A (2016). Quality of life in medical students with Internet addiction. Acta Med Iran.

[64] Mei S, Yau YHC, Chai J, Guo J, Potenza MN, Chan RCK (2016). Effects of internet addiction on family relationships among Chinese adolescents and young adults. Cyberpsychol Behav Soc Netw.

[65] Peterka-Bonetta J, Sindermann C, Sha P, Zhou M, Montag C (2019). The relationship between Internet use disorder, depression and burnout among Chinese and German college students. Addict Behav.

[66] Berte DZ, Mahamid FA, Affouneh S (2021). Internet addiction and perceived self-efficacy among university students. Int J Ment Heal Addict.

[67] Nelsen SK, Kayaalp A, Page KJ. Perfectionism, substance use, and mental health in college students: a longitudinal analysis. J Am Coll Health. 2023;71(1):257–65. 10.1080/07448481.2021.1891076. 10.1080/07448481.2021.1891076 33759725

[68] Noori MA, Saadi S, Al-Zu'bi AH (2022). The impact of social media on the EFL learning process: a case study of Jordanian students. Int J Engl Linguist.

[69] An H, Williams MK (2010). Identity construction and language learning: a case study of a Korean student's blogs. Comput Compos.

[70] Mitchell K (2012). Writing and social media: The benefits and drawbacks of using Facebook and Twitter in the classroom. J Educ Technol Dev Exch.

[71] Creswell JW (2014). Research design: Qualitative, quantitative, and mixed methods approach.

[72] Zhao N (2007). A study of high school students’ English learning anxiety. Asian-EFL J.

[73] Dai XY (2010). Handbook of Commonly Used Psychological Assessment Scales.

[74] Berg BL (2001). Qualitative research methods for the social sciences.

[75] Yang D, Wu W (2012). Collaborative learning using social media tools in EFL context. Engl Lang Teach.

[76] Yang CC, Wu CH (2012). The effects of social media on college students. J Educational Technology Development and Exchange.

[77] Simsek H, Sali J (2014). Factors affecting social media usage and social media addiction in university students. J Educ Sci Res.

[78] Wang Q, Vasquez C (2014). Facebook as a formal instructional environment. Br J Edu Technol.

[79] Jafari M, Janatolmakan M, Khubdast S, Azizi M, Khatony A (2022). The Relationship of Internet Abusive Use with Academic Burnout and Academic Performance in Nursing Students. Available at:.

[80] Kumar S, Tiwari SC (2015). Internet addiction among students and its relation with academic performance. J Indian Acad Appl Psychol.

[81] Salih ZA, Elsaid RA (2018). Social media use and academic achievement among university students: a pilot study. J Educ Pract.

[82] Bani-Hani AF, Al-Shboul M, AL Zboon E (2014). The impact of using social media on EFL students’ achievement. Engl Lang Teach.

[83] Haand FA, Shuwang M (2020). Social media addiction among university students: A case study of Babcock University. Int J Inform Sci Manag.

[84] Kirkgöz Y (2011). The effects of social network sites on English language learning: a case study in a Turkish setting. Turkish Online J Educ Technol.

[85] Haidari S, Khodabakhshzadeh H, Ghadiri V (2020). The impact of using social networks on EFL learners' critical thinking skills and literacy proficiency. Int J Emerg Technol Learn.

[86] Rodriguez JE (2011). Social media use in higher education: Key areas to consider for educators. J Online Learn Teach.

[87] Mwalimu DM, Kinyua JK, Mwangi WK (2018). Social media usage among university students in Kenya: a survey of Twitter use at Kenyatta University. Int J Innov Sci Res.

[88] Esteve F, Adell J, Gisbert M (2017). An analysis of teachers' use of Facebook for educational purposes at the university level. Teach Teach Educ.

[89] Khan NA, Azhar M, Rahman MN, Akhtar MJ (2022). Scale development and validation for the usage of social networking sites during COVID-19. Technol Soc.

[90] Bresó E, Salanova M, Schaufeli W (2007). In Search of the “Third Dimension” of Burnout: Efficacy or Inefficacy?. Appl Psychol.

[91] Woods HC, Scott H (2016). #Sleepyteens: Social media use in adolescence is associated with poor sleep quality, anxiety, depression and low self-esteem. J Adolesc.

[92] Fincham FD, Cui M, Braithwaite SR, Pasley K (2009). Forgiveness and psychological capital in college: counteracting negative effects of stress on retention. J Couns Dev.

