# Supplementary material for: EFL learners’ problematic use of social media usage, classroom anxiety, perfectionism, and language attainment: correlations and perceptions
Source: BMC Psychol. 2023 Dec 18;11:443. doi: 10.1186/s40359-023-01419-5 (PMC10726621; doi:10.1186/s40359-023-01419-5)
Supplement: Supplementary file 1 — Additional file 1. [file 40359_2023_1419_MOESM1_ESM.docx]

Appendix

Interview Checklist

1. In what ways do you think social media applications like Telegram and WhatsApp can positively impact your language achievement?
2. Have you ever experienced any negative effects on your language achievement as a result of using social media applications for language learning? If so, can you describe them?
3. How do you think social media applications can affect your mental health, positively or negatively?
4. Have you ever experienced any negative effects on your mental health as a result of using social media applications frequently? If so, can you describe them?
5. Have you noticed any changes in your anxiety levels as a result of using social media applications frequently? If so, can you describe them?
6. How do you think social media applications can contribute to academic burnout?
7. Have you ever experienced academic burnout as a result of using social media applications frequently? If so, can you describe that experience?
8. In your opinion, what are the most problematic and non-problematic social media applications for language learning, mental health, anxiety level, and academic burnout? Can you explain why?
